# Probing the biophysical constraints of SARS-CoV-2 spike N-terminal domain using deep mutational scanning

**DOI:** 10.1126/sciadv.add7221

**Published:** 2022-11-23

**Authors:** Wenhao O. Ouyang, Timothy J.C. Tan, Ruipeng Lei, Ge Song, Collin Kieffer, Raiees Andrabi, Kenneth A. Matreyek, Nicholas C. Wu

**Affiliations:** ^1^ Department of Biochemistry, University of Illinois at Urbana-Champaign, Urbana, IL 61801, USA.; ^2^ Center for Biophysics and Quantitative Biology, University of Illinois at Urbana-Champaign, Urbana, IL 61801, USA.; ^3^ Department of Immunology and Microbiology, The Scripps Research Institute, La Jolla, CA 92037, USA.; ^4^ IAVI Neutralizing Antibody Center, The Scripps Research Institute, La Jolla, CA 92037, USA.; ^5^ Consortium for HIV/AIDS Vaccine Development (CHAVD), The Scripps Research Institute, La Jolla, CA 92037, USA.; ^6^ Department of Microbiology, University of Illinois at Urbana-Champaign, Urbana, IL 61801, USA.; ^7^ Department of Pathology, Case Western Reserve University School of Medicine, Cleveland, OH 44106, USA.; ^8^ Carl R. Woese Institute for Genomic Biology, University of Illinois at Urbana-Champaign, Urbana, IL 61801, USA.; ^9^ Carle Illinois College of Medicine, University of Illinois at Urbana-Champaign, Urbana, IL 61801, USA.

## Abstract

Increasing the expression level of the SARS-CoV-2 spike (S) protein has been critical for COVID-19 vaccine development. While previous efforts largely focused on engineering the receptor-binding domain (RBD) and the S2 subunit, the amino-terminal domain (NTD) has been long overlooked because of the limited understanding of its biophysical constraints. In this study, the effects of thousands of NTD single mutations on S protein expression were quantified by deep mutational scanning. Our results revealed that in terms of S protein expression, the mutational tolerability of NTD residues was inversely correlated with their proximity to the RBD and S2. We also identified NTD mutations at the interdomain interface that increased S protein expression without altering its antigenicity. Overall, this study not only advances the understanding of the biophysical constraints of the NTD but also provides invaluable insights into S-based immunogen design.

## Teaser

A survey on thousands of SARS-CoV-2 S protein NTD mutations reveals a complementary strategy for immunogen optimization.

## INTRODUCTION

The emergence of severe acute respiratory syndrome coronavirus-2 (SARS-CoV-2) has led to the coronavirus disease 2019 (COVID-19) pandemic (*1*, *2*). As the major antigen of SARS-CoV-2, spike (S) glycoprotein plays a critical role in facilitating virus entry (*3*, *4*). Therefore, antibodies to SARS-CoV-2 S are often neutralizing (*5*, *6*). SARS-CoV-2 S protein consists of an N-terminal S1 subunit, which is responsible for engaging the host receptor angiotensin-converting enzyme 2 (ACE2) via the receptor-binding domain (RBD), as well as a C-terminal S2 subunit, which mediates virus-host membrane fusion (*4*, *7*, *8*). The S1 subunit also contains an N-terminal domain (NTD) in addition to the RBD (*4*, *7*). While the RBD is generally considered to be immunodominant, the NTD is also a target of neutralizing antibodies (*9*–*11*). Structural studies revealed the presence of an antigenic supersite on the NTD that is frequently mutated in SARS-CoV-2 variants of concern (VOCs) (*12*–*17*). Amino acid mutations and indels rapidly accumulate within the NTD during the evolution of SARS-CoV-2 in human, at least partly due to the immune selection pressure (*18*). On the other hand, antibodies to NTD epitopes that are conserved across VOCs have also been identified (*16*, *19*). Despite the importance of NTD in immune response against SARS-CoV-2, the biophysical constraints of NTD remain largely elusive.

COVID-19 vaccines, including both recombinant protein–based and mRNA-based, are proven to be highly protective against SARS-CoV-2 infection (*20*–*23*). There is an inverse relationship between the production yield and cost of recombinant protein–based COVID-19 vaccines, such as that from Novavax, which showed promising results in phase 3 clinical trials (*22*), as well as others that are in earlier phases of clinical trials (*24*). High protein expression level is also believed to be critical for the effectiveness of mRNA vaccines (*25*). As a result, identifying mutations that increase S protein expression are crucial for optimizing COVID-19 vaccines. While most studies focused on mutating the S2 subunit and the RBD to increase S protein expression (*7*, *26*–*29*), little effort has been spent on NTD because of the lack of understanding of its biophysical properties.

Phenotypes of numerous mutations can be measured in a massively parallel manner using deep mutational scanning, which combines saturation mutagenesis and next-generation sequencing (*30*). Previous studies have applied deep mutational scanning to evaluate the effects of RBD mutations on protein expression, ACE2-binding affinity, and antibody escape (*31*–*36*). Although deep mutational scanning of the RBD provided important insights into immunogen design and SARS-CoV-2 evolution (*29*, *31*, *32*, *35*, *36*), similar studies on other regions of the S protein have not yet been carried out.

Here, we used deep mutational scanning to quantify the effects of thousands of NTD single mutations on S protein expression. One notable observation was that NTD residues, unlike RBD residues, showed a weak correlation between mutational tolerability and relative solvent accessibility (RSA). Instead, the mutational tolerability of NTD residues strongly correlated with their distance to RBD and S2. Residues S50 and G232 were two exceptions, in which they were proximal to S2 and RBD, respectively, and yet had a high mutational tolerability. Subsequently, we functionally characterized two mutations that increased S protein expression, namely, S50Q and G232E. These results have important implications toward understanding NTD evolution and S-based immunogen design.

## RESULTS

### Most NTD mutations have minimal impact on S protein expression

To study how SARS-CoV-2 S protein expression is influenced by NTD mutations, we created a mutant library that contained all possible single amino acid mutations across residues 14 to 301 of the S protein. Each of these 288 residues was mutated with the choice of all 19 other amino acids and the stop codon, leading to a mutant library with 5760 single amino acid mutations. We used a cassette-based polymerase chain reaction (PCR) strategy that was designed to avoid potential off-target errors and generation of double mutants during the library construction process (fig. S1). As a quality control, Sanger sequencing was performed on 10 selected colonies following the construction of the mutant library. All of them contained a single mutation as expected. The mutant library was expressed using the human embryonic kidney (HEK) 293T landing pad cell system such that each transfected cell stably expressed only one mutant (*37*, *38*). Fluorescence-activated cell sorting (FACS) was then performed using the human anti-S2 antibody CC40.8 (*39*), with phycoerythrin (PE) anti-human immunoglobulin G (IgG) Fc as the secondary antibody. Four separated gates were set up on the basis of the PE signals, each covering 25% of the entire population (fig. S2). The frequency of each mutant among the entire population was calculated (see Materials and Methods), and a cutoff of 0.0075% was set up to filter out mutants with potentially noisy measurements. Among the 5760 missense and nonsense mutations, 3999 (69%) of them satisfied the frequency cutoff for downstream analysis. Notably, the design of our mutant library adopted an internal barcoding strategy that uses synonymous mutations to facilitate sequencing error correction (*40*). As described previously (*41*), the expression score of each mutation was calculated on the basis of their frequency in each of the four gates and normalized such that the average expression score of silent mutations was 1 and that of nonsense mutations was 0. Blocks of missing sites could be observed periodically across the NTD, potentially due to the locations of NNK codons in our cassette PCR design (fig. S3).

To evaluate the quality of the deep mutational scanning results, we assessed the expression score distributions of missense, nonsense, and silent mutations (fig. S4A). The difference between the expression scores of silent mutations and nonsense mutations was apparent and significant (*P* = 6 × 10^−166^), which validated the selectivity of the deep mutational scanning experiment. Silent mutation and missense mutations had similar expression scores, although the difference is statistically significant (*P* = 2 × 10^−5^), indicating that most amino acid mutations in the NTD did not affect S protein expression. While dead cells could potentially increase the noise of the deep mutational scanning results, this effect seemed to be relatively minor in our experiment (fig. S5). In addition, a Pearson correlation of 0.53 was obtained between the expression scores of each mutant from two independent biological replicates (fig. S4B), demonstrating the reproducibility of the deep mutational scanning experiment.

**Fig. 1.**
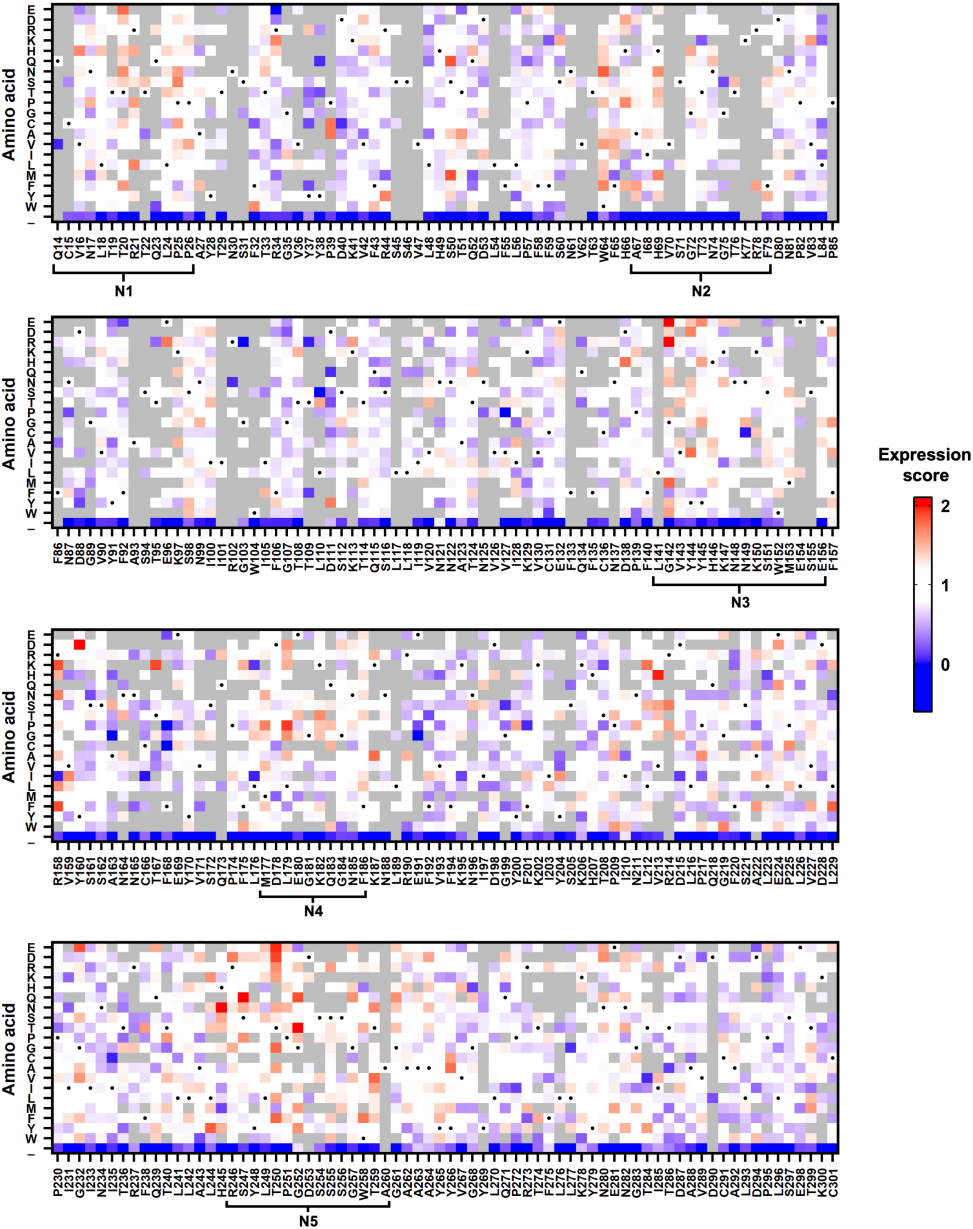
Effects of NTD single mutations on S protein expression. The expression scores of individual NTD mutations are shown as a heatmap. *X* axis represents the residue position. *Y* axis represents different amino acids and the stop codon (_). Amino acids corresponding to the wild-type (WT) sequence are indicated by the black dots. Mutations with a total frequency of <0.0075% were excluded from the analysis and shown in gray. Regions corresponding to the N1-N5 loops were defined as previously described (*12*).

To summarize the expression scores for individual mutations, a heatmap was generated (Fig. 1). We noticed that high-expressing mutations were enriched within the five NTD loop regions (fig. S7A) (*12*). High-expressing mutations were also found in residues outside of the loop regions, such as residues S50 and G232. This observation shows that some NTD mutations can improve the expression of S protein.

### Mutational tolerability has minimal correlation with solvent accessibility

While some residues were enriched in high-expression mutations (see above), others were enriched in low-expression mutations (e.g., residues D40, L84, and N234; Fig. 1). Consequently, we aimed to identify the biophysical determinants of mutational tolerability in terms of S protein expression. For each residue, we defined the mutational tolerability as the mean expression score of mutations. A higher mutational tolerability would indicate the enrichment of high-expressing mutations at the specified residue. In contrast, a lower mutational tolerability would indicate the enrichment of low-expressing mutations at the specified residue. A total of 243 NTD residues had six or more mutations with expression score available and were included in this analysis. Notably, a Pearson correlation of 0.68 was obtained between the mutational tolerability values of each position from the two independent biological replicates (fig. S4C).

**Fig. 2.**
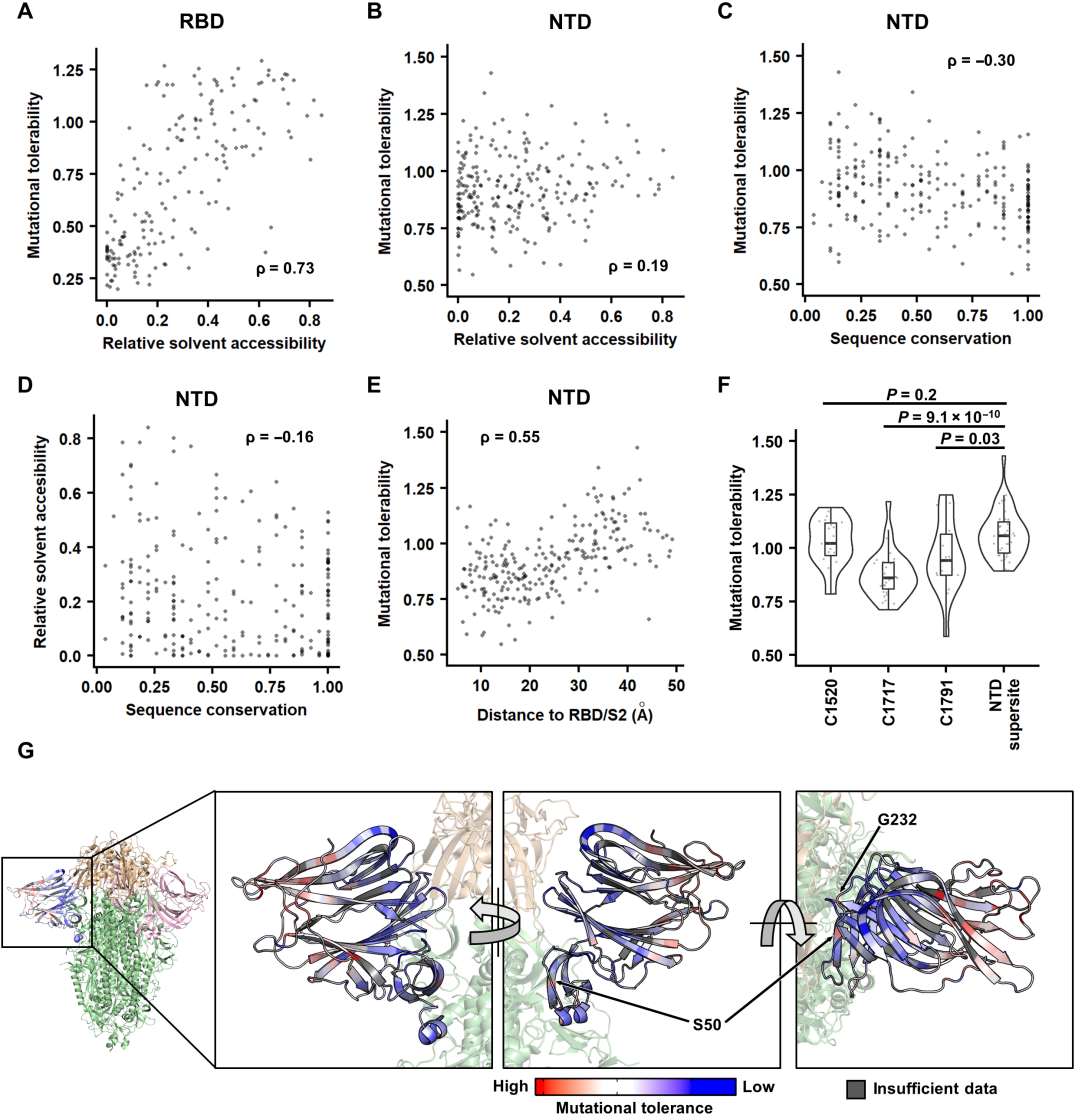
Identifying the biophysical determinants of mutational tolerability. (**A** and **B**) The relationship between RSA and the mutational tolerability is shown for (A) RBD and (B) NTD. The deep mutational scanning data on RBD expression were from a previous study (*42*). (**C** and **D**) Relationship between sequence conservation among 27 sarbecovirus strains (table S6) and (C) the mutational tolerability or (D) RSA of each NTD residue. (**E**) Relationship between the distance to RBD/S2 and the mutational tolerability of each NTD residue. (A to E) Each data point represents one residue. The Spearman’s rank correlation coefficient (ρ) is indicated. (**F**) The mutational tolerability of residues within the cross-neutralizing NTD antibody epitopes (C1520, C1717, and C1791) (*16*) is compared to that within the antigenic supersite (*14*) using a violin plot. Each data point represents one residue. *P* values were computed by two-tailed *t* test. (**G**) The mutational tolerability of each NTD residue is projected on one NTD of the S trimer structure [Protein Data Bank (PDB) 6ZGE (*45*) and PDB 7B62 (*46*)]. Red indicates residues with higher mutational tolerability, while blue indicates residues with lower mutational tolerability. Residues with insufficient data to calculate mutational tolerability are colored in gray. Two residues of interests, namely, S50 and G232, are indicated. RBDs are colored in wheat, the two other NTDs are in pink, and the rest of the S1 and S2 subunits are in green.

First, we investigated whether a correlation existed between the mutational tolerability and RSA. Because buried residues are typically important for protein folding stability, residues with a lower RSA are generally expected to have a lower mutational tolerability. For example, previous deep mutational scanning studies on the RBD have shown a decent correlation between RSA and mutational tolerability (Spearman correlation = 0.73; Fig. 2A) (*34*, *42*). In contrast, the mutational tolerability of NTD residues had a much weaker correlation with RSA (Spearman correlation = 0.19; Fig. 2B). These observations indicate that the folding stability of NTD does not have a strong influence on its mutational tolerability and, hence, the S expression level. Alternatively, it is possible that some mutations can destabilize NTD, but NTD instability does not affect the S expression level.

To investigate whether the mutational tolerability correlated with sequence conservation, we then analyzed the NTD sequences of 27 sarbecovirus strains, including SARS-CoV-2. Less conserved residues tended to have a higher mutational tolerability, while more conserved residues tended to have a lower mutational tolerability, although the correlation was not strong (Spearman correlation = −0.30; Fig. 2C). In comparison, the correlation between sequence conservation and RSA was even weaker (Spearman correlation = −0.16; Fig. 2D).

### Mutational tolerability correlates with distance to RBD/S2

We further calculated the distance from each NTD residue to RBD/S2 of the S protein. A positive correlation was observed between the mutational tolerability and the distance to RBD/S2 (Spearman correlation = 0.55; Fig. 2E). In other words, the more distant an NTD residue was from the RBD/S2, the higher the mutational tolerability was. This correlation was apparent when the mutational tolerability of each NTD residue was projected on the S protein structure (Fig. 2G). Consistently, the epitopes of two cross-neutralizing antibodies, namely, C1717 and C1791, were significantly closer to RBD/S2 (*P* ≤ 1 × 10^−4^) and had lower mutational tolerability (*P* ≤ 0.03) when compared to the rapidly evolving NTD antigenic supersite (Fig. 2F and fig. S7B) (*14*, *16*, *43*).

### Naturally circulating NTD indel sites have significantly higher mutational tolerability

**Fig. 3.**
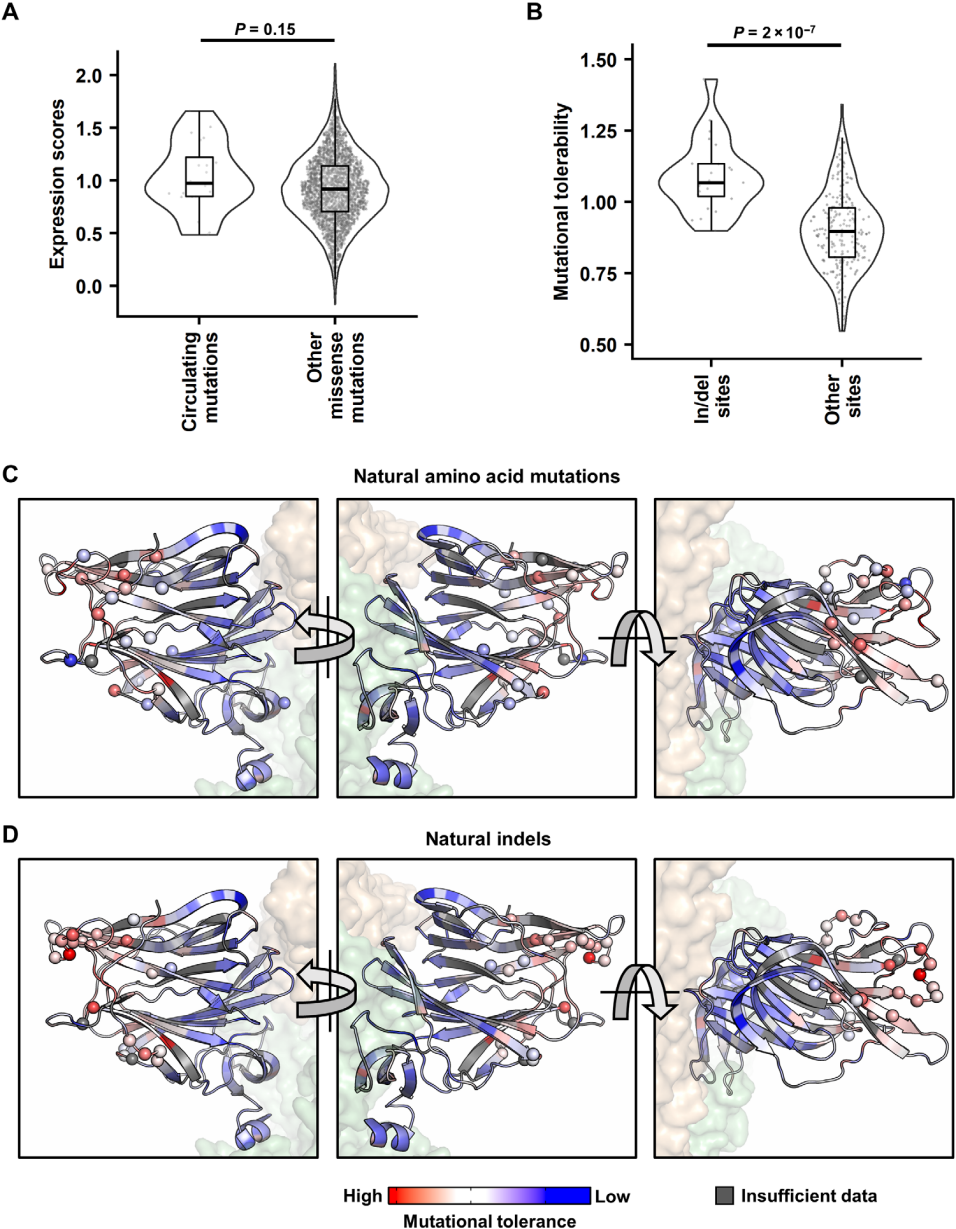
Analysis of naturally circulating NTD mutations. (**A**) Expression scores of naturally occurring NTD mutations and all other missense mutations. (**B**) Mutational tolerability of naturally occurring indel sites and all other sites. *P* values were computed by two-tailed *t* test. (**C** and **D**) Missense mutations (C) and indel sites (D) observed in 17 SARS-CoV-2 major variants are labeled as spheres on one NTD of the S trimer structure [PDB 6ZGE (*45*) and PDB 7B62 (*46*)]. Red indicates residues with higher mutational tolerability, while blue indicates residues with lower mutational tolerability. Residues with insufficient data to calculate mutational tolerability are colored in gray. The neighboring RBDs are colored in wheat, and the rest of the S1 and S2 subunits shown are colored in green.

We then examined the naturally occurring NTD mutations and indels observed among 17 SARS-CoV-2 major variants using our deep mutational scanning data. Among these 17 variants, there are 25 different amino acid mutations and 25 indel sites relative to the ancestral strain. Among the 25 amino acid mutations and 25 indel sites, 20 and 23, respectively, have available expression scores and site-wise mutational tolerability in our dataset. The expression score distribution of the 20 natural amino acid mutations was similar to the rest of the missense mutations (*P* = 0.15; Fig. 3A). In contrast, the 23 natural indel sites had significant higher mutational tolerability than the other NTD residues (*P* = 2 × 10^−7^; Fig. 3B). This observation is consistent with the enrichment of natural indels in the five NTD loops, which have high mutational tolerability (Fig. 3, C and D).

### Two buried NTD mutations increase S protein expression

While NTD residues adjacent to RBD/S2 typically had a low mutational tolerability, S50 and G232 were two exceptions (Fig. 2G). For example, mutations S50G and G232E had a high expression score in our deep mutational scanning results. To validate this finding, we used the same landing pad system to construct HEK293T cell lines that stably expressed S50Q, G232E, and S50Q/G232E double mutant. As quantified by flow cytometry analysis (Fig. 5A and fig. S8), the mean fluorescence intensity (MFI) of S50Q and G232E increased from wild type (WT) by 1.7-fold (*P* = 0.002) and 1.5-fold (*P* = 9 × 10^−4^), respectively, whereas that of S50Q/G232E increased by 2.5-fold (*P* = 2 × 10^−6^). Although fold change in MFI is unlikely to linearly correlate with the fold change in protein synthesis, such increase in MFI shows that S50Q, G232E, and S50Q/G232E double mutant have increased surface expression compared to WT.

**Fig. 4.**
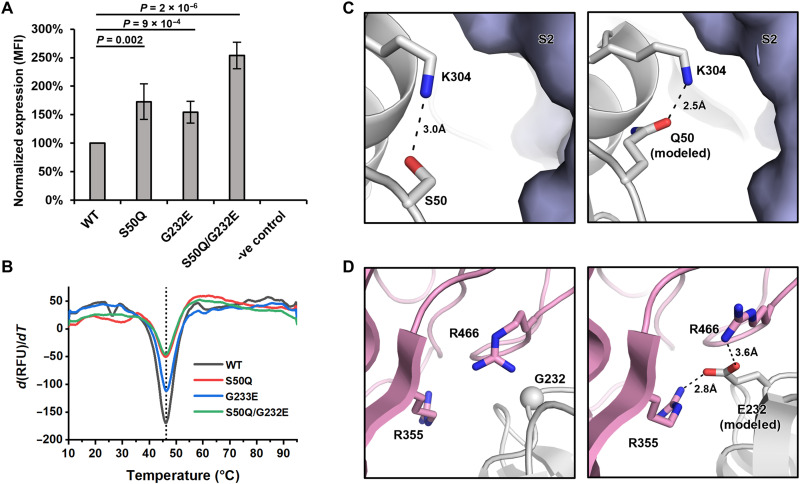
S50Q and G232E at the interdomain interface increase S protein expression. (**A**) Cell surface S protein expression of WT and NTD mutants was quantified using flow cytometry analysis with CC40.8 as the primary antibody. Untransfected HEK293T landing pad cells were used as a negative control (-ve control). S protein expression level was defined as the MFI of the positive gated population. S protein expression level was normalized to WT. The error bar indicates the SD of six independent biological replicates. *P* values were computed by two-tailed *t* test. (**B**) Thermostability of WT S protein and selected NTD mutants was measured using differential scanning fluorimetry. The black vertical dotted line indicates the melting temperature of WT (*T*_m_ = 46.2°C). (**C** and **D**) Rosetta-based structural modeling of (C) S50Q and (D) G232E was performed using the structure of S protein (PDB 6ZGE) (*46*). The three protomers of the S protein are colored in white, light blue, and pink. Potential interactions are represented by black dashed lines with distance labeled.

**Fig. 5.**
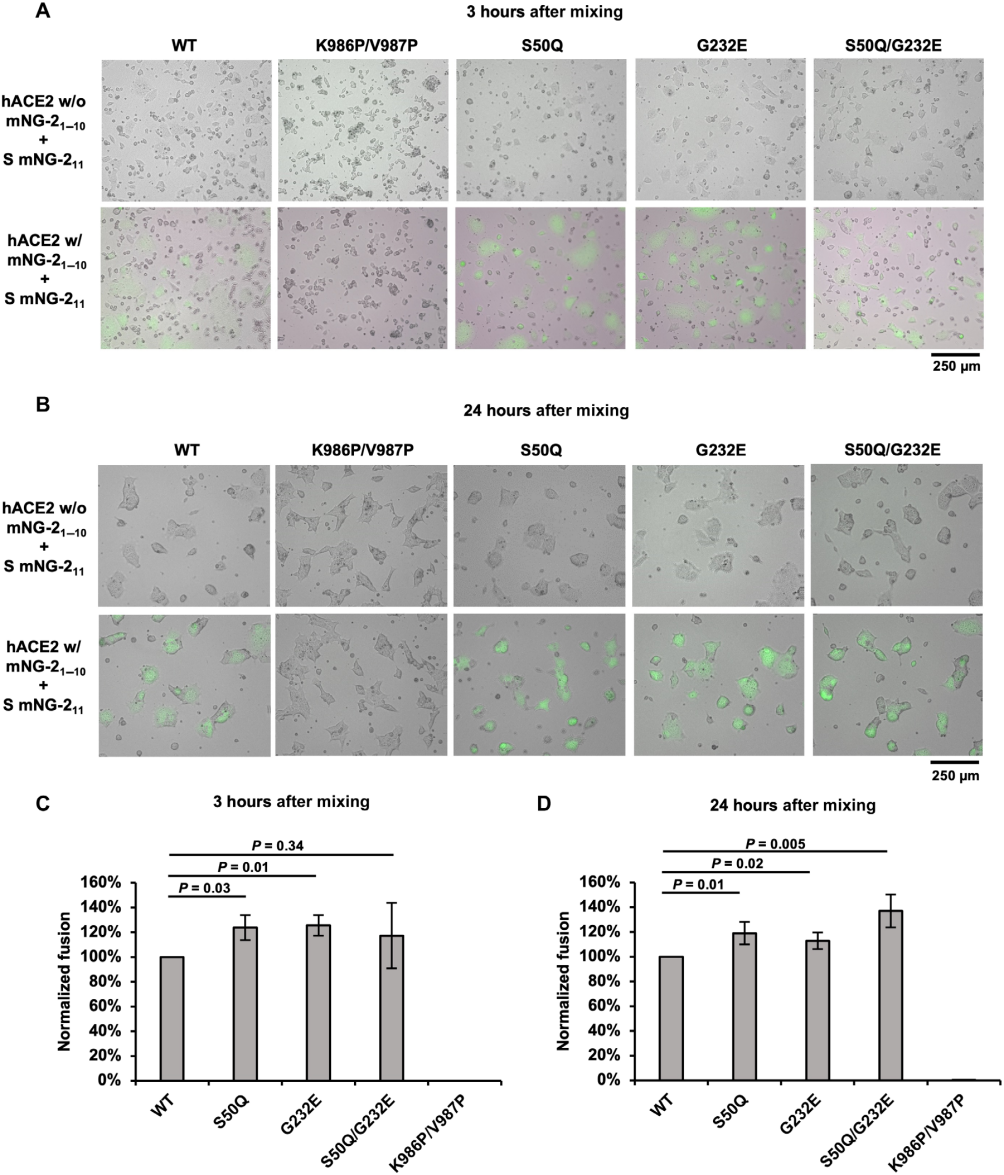
S50Q, G232E, and S50Q/G232E do not diminish fusion activity. (**A** and **B**) Fluorescence microscopy analysis of the fusion events at (A) 3-hour and (B) 24-hour postmixing of cells expressing S and mNG2_11_ (S cells) and cells expressing hACE2 and mNG2_1–10_ (hACE2 cells). Cells with green fluorescence signals are the fused cells. Scale bars are shown at the bottom right corner. (**C** and **D**) Fusion activity of WT and selected NTD mutants at (C) 3-hour and (D) 24-hour postmixing was quantified using flow cytometry analysis. Fusion activity was normalized to WT. The error bar indicates the SD of at least four biological replicates. *P* values were computed by two-tailed *t* test.

Subsequently, we examined the natural occurrences of S50Q and G232E mutations. Both S50Q and S232E rarely occur in circulating SARS-CoV-2. Among over 10 million NTD sequences on Global Initiative for Sharing Avian Influenza Data (GISAID) (*44*), only 22 and 3 sequences contain S50Q and G232E, respectively. To probe the structural impact of S50Q and G232E, we analyzed their local environments on the structure of S protein and performed structural modeling using Rosetta (Fig. 5, C and D) (*45*–*47*). S50 forms a hydrogen bond with K304 and is proximal to the S2 subunit. Structural modeling showed that S50Q not only is able to maintain the hydrogen bond with K304 but also strengthens the van der Waals interaction between the NTD and S2 by pushing K304 toward S2 from the adjacent protomer (Fig. 5C). G232 is proximal to a positively charged region on the RBD that is featured by R355 and R466 (Fig. 5D). Structural modeling suggested that G232E could form favorable electrostatic interactions with both R355 and R466. We further recombinantly expressed these mutants and tested their thermostability using a thermal shift assay (Fig. 5B). Notably, all the recombinantly expressed S proteins contained K986P/V987P mutations in the S2 subunit, which are known to stabilize the prefusion conformation and increase expression (*26*, *48*). The melting temperatures of WT and NTD mutants were almost identical at a *T*_m_ of 46°C to 46.5°C. These observations indicate that despite both S50Q and G232E improve the interaction between NTD and the rest of the S protein, they have minimal impact on the global folding stability of the S protein. Notably, all mutants increased the yield of the soluble recombinantly expressed S protein compared to WT (fig. S6), although the rank order was different from that of the membrane-bound form (Fig. 4A and fig. S6).

### S50Q and G232E have minimal effects on the fusion activity and antigenicity

**Fig. 6.**
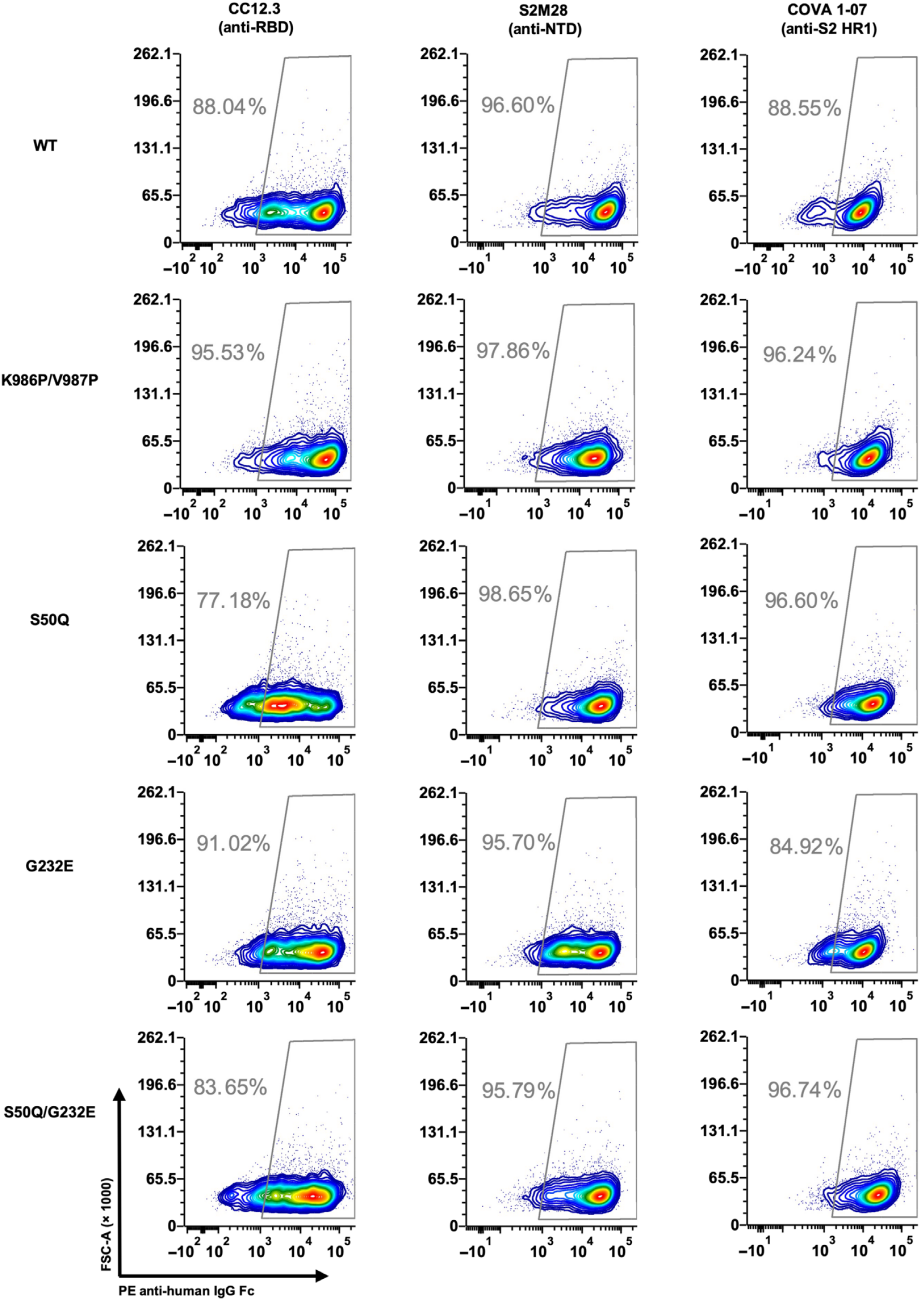
S50Q, G232E, and S50Q/G232E do not alter antibody binding. Three antibodies targeting different domains on S were tested for binding to cells expressing WT, K986P/V987P, S50Q, G232E, or S50Q/G232E S protein. Binding was measured by flow cytometry analysis. Gating was set up using untransfected HEK293T landing pad cells, which served as a negative control (fig. S10).

To understand the functional consequences of S50Q and G232E, we further tested whether S50Q, G232E, and S50Q/G232E exhibited a change in fusion activity compared to WT. A fluorescence-based cell-cell fusion assay that relied on the split mNeonGreen2 (mNG2) (*49*) was performed (see Materials and Methods; fig. S9). Briefly, HEK293T landing pad cells that expressed human ACE2 (hACE2) and mNG2_1–10_ were mixed with HEK293T landing pad cells that expressed S proteins and mNG2_11_. Green fluorescence due to mNG2 complementation was generated when fusion between the two cell lines occurred. Fluorescence microscopy analysis showed that all mutants facilitated hACE2-mediated fusion (Fig. 6, A and B). Consistently, flow cytometry analysis at both 3-hour and 24-hour postmixing indicated that none of the tested mutants diminished the fusion activity when compared to WT (Fig. 6, C and D). At 3-hour postmixing, both S50Q (24%, *P* = 0.03) and G232E (25%, *P* = 0.01) showed mild, yet significant, increases in fusion activity compared to WT. Similarly, at 24-hour postmixing, S50Q (19%, *P* = 0.01), G232E (13%, *P* = 0.02), and S50Q/G232E double mutant (37%, *P* = 0.005) all showed an increase in fusion activity compared to WT. Such a mild increase in fusion activity may simply be attributed to the higher expression level of the mutants. However, because the relationship between protein expression level and fusion activity remained elusive, we are unable to assess the fusion activity per S protein molecule based on the current data. Negative control cells expressing the K986P/V987P double mutant, which is known to stabilize the prefusion form of the S protein (*26*, *48*), did not show any fusion activity (Fig. 5, A to D). Overall, our results demonstrated that both S50Q and G232E did not affect the fusogenic capabilities of the S protein.

We then proceeded to investigate whether S50Q, G232E, and S50Q/G232E alter the antigenicity of the S protein. The binding of three antibodies targeting different domains of the S protein was tested, namely, CC12.3 (anti-RBD) (*50*), S2M28 (anti-NTD) (*14*), and COVA1-07 (anti-S2) (*51*). Notably, S2M28 is an NTD supersite–targeting antibody. Flow cytometry analysis showed that all three antibodies bound to the tested mutants at a similar level to WT (Fig. 6 and fig. S10), indicating that S50Q, G232E, and S50Q/G232E did not alter the structural conformation and antigenicity of the S protein.

## DISCUSSION

S protein is central to the research of SARS-CoV-2 evolution and COVID-19 vaccines (*50*, *52*–*54*). While both the RBD and the NTD on the S protein are targets of neutralizing antibodies and are involved in the antigenic drift of SARS-CoV-2 (*43*, *55*–*61*), the NTD often receives less attention than does the RBD. Using deep mutational scanning, this study shows that many NTD mutations at buried residues do not affect S protein expression. At the same time, the closer an NTD mutation is to RBD/S2, the more likely it is detrimental to S protein expression. These observations imply that for optimum S protein expression, the structural stability at the NTD-RBD and the NTD-S2 interfaces is more critical than the folding stability of the NTD. Our results also at least partly explain why the N1 to N5 loops, which contain the NTD antigenic supersite (*62*) and are far from the NTD-RBD/S2 interfaces, are highly diverse among SARS-CoV-2 variants and sarbecovirus strains. Overall, this study provides crucial biophysical insights into the evolution of the NTD.

NTD mutations S50Q and G232E, which locate at the interdomain interface and increase S protein expression, represent another important finding of this study. Engineering high-expressing S protein can lower the production cost of recombinant COVID-19 vaccine and may improve the effectiveness of mRNA vaccines (*25*). Similar to certain previously characterized mutations in the S2 (*26*, *27*), S50Q and G232E in the NTD increase the expression yield of the S protein without changing its *T*_m_. Consistently, a recent study showed that NTD mutations in BA.1 improve the expression of S protein without increasing its thermostability (*63*). Furthermore, S50Q and G232E are not solvent-exposed on the S protein surface and do not seem to alter the antigenicity of the S protein. Notably, according to our deep mutational scanning data, S50Q and G232E are just two of many mutations that enhance S protein expression. Therefore, although most studies on S-based immunogen design focus on the mutations in the RBD and S2 (*7*, *26*–*29*), our results suggest that mutations in NTD can provide a complementary strategy.

We acknowledge that the S protein expression level does not necessarily correlate with virus replication fitness. For example, NTD mutations that do not affect the S protein expression may be detrimental to the replication fitness of SARS-CoV-2 due to the negative impact on NTD functionality. While the functional importance of the NTD in natural infection remains largely unclear, NTD has been proposed to facilitate virus entry by interacting with DC-SIGN, L/SIGN, AXL, ASGR1, and KREMEN1 (*64*–*66*). Studies have also shown that the NTD can allosterically evade antibody binding by interacting with a heme metabolite (*46*) and modulate the efficiency of virus-host membrane fusion (*67*, *68*). To fully comprehend the biophysical constraints of NTD, future studies should systematically investigate how different NTD mutations affect virus replication fitness.

## MATERIALS AND METHODS

### Construction of the NTD mutant library

SARS-CoV-2 S NTD mutant library was constructed based on the HEK293T landing pad system (*37*, *38*). The template for constructing the NTD mutant library was a plasmid that encoded (from 5′ to 3′) an attB site, a codon-optimized SARS-CoV-2 S (GenBank ID: NC_045512.2) with the PRRA motif in the furin cleavage site deleted, an internal ribosome entry site (IRES), and a puromycin resistance marker. This plasmid was used as a PCR template to generate a linearized vector and a library of mutant NTD inserts. The linearized vector was generated using 5′-TGCTCGTCTCTACAACTCCGCCAGCTTCAGCACC-3′ and 5′-TGCTCGTCTCTTCACTGGCCGTCGTTTTACAACG-3′ as primers. Inserts were generated by two separate batches of PCRs to cover the entire NTD. The first batch of PCRs consisted of 36 reactions, each containing one cassette of forward primers and the universal reverse primer 5′-TGCTCGTCTCGTTGTACAGCACGGAGTAGTCGGC-3′. Each cassette contained an equal molar ratio of eight forward primers that had the same 21 nucleotides (nt) at the 5′ end and 15 nt at the 3′ end. Each primer within a cassette was also encoded with an NNK (N: A, C, G, T; K: G, T) sequence at a specified codon positions for saturation mutagenesis. In addition, each primer also carried unique silent mutations (also known as synonymous mutations) to help distinguish between sequencing errors and true mutations in downstream sequencing data analysis as described previously (*40*). The forward primers, named as CassetteX_N (X: cassette number, N: primer number), are listed in table S1. The second batch of PCR consisted of another 36 PCRs, each with a universal forward primer 5′-TGCTCGTCTCAGTGAATTGTAATACGACTCACTA-3′ and a unique reverse primer as listed in table S2. Subsequently, 36 overlapping PCRs were performed using the universal forward and reverse primers, as well as a mixture of 10 ng each of the corresponding products from the first and second batches of PCR. The 36 overlap PCR products were then mixed at equal molar ratio to generate the final insert of the NTD mutant library. All PCRs were performed using PrimeSTAR Max polymerase (Takara Bio, catalog no. R045B) per the manufacturer’s instruction, followed by purification using the Monarch Gel Extraction Kit (New England Biolabs, catalog no. T1020L). The final insert and the linearized vector were digested by BsmBI-v2 (New England Biolabs, catalog no. R0739L) and ligated using T4 DNA Ligase (New England Biolabs, catalog no. M0202L). Ligation product was purified by the PureLink PCR Purification Kit (Thermo Fisher Scientific, catalog no. K310002) and then transformed into MegaX Dh10B T1R cells (Thermo Fisher Scientific, catalog no. C640003). At least half a million colonies were collected. Plasmid mutant library was purified from the bacteria colonies using the PureLink HiPure Plasmid Midiprep Kit (Invitrogen, catalog no. K210005). All primers in this study were ordered from Integrated DNA Technologies.

### Construction of stable cell lines using HEK293T landing pad cells

HEK293T landing pad cells (*37*, *38*) were used to display the NTD mutant library for deep mutational scanning. Landing pad cells were maintained using complete growth medium consisting of Dulbecco’s modified Eagle’s medium (DMEM; Corning), 10% (v/v) fetal bovine serum (FBS; VWR), penicillin-streptomycin (Gibco), nonessential amino acid (Gibco), and doxycycline (2 μg/ml). Plasmid (1.2 μg) was transfected into 6 × 10^5^ landing pad cells. For the deep mutational scanning experiment, eight transfection reactions were carried out in parallel to minimize loss of mutant diversity at the transfection step. Transfected cells were then incubated at 37°C with 5% CO_2_. After 48 hours, 10 nM AP1903 was supplemented to carry out negative selection. At 72 hours after the negative selection, positive selection antibiotic [puromycin (1 μg/ml) for NTD cell lines or hygromycin (100 μg/ml) for hACE2 cell lines] was supplemented to the medium to carry out positive enrichment of cells with successful recombination. Constructed cell lines would remain in the complete growth medium supplemented with doxycycline and the positive selection antibiotics.

### Sorting the NTD mutant library based on S protein expression level

Four T-75 flasks (Corning) that were 90% confluent with cells that carried the NTD mutant library were washed with 1× phosphate-buffered saline (PBS), harvested with warm versene, and pelleted via centrifugation at 300*g* for 5 min at room temperature. Cells were then resuspended in FACS buffer [2% (v/v) FBS, 5 mM EDTA in DMEM supplemented with glucose, l-glutamine, and Hepes but without phenol red (Gibco)]. Subsequently, cells were incubated with CC40.8 (5 μg/ml) at 4°C with gentle shaking for 1 hour. Cells were washed once with ice-cold FACS buffer and incubated with PE anti-human IgG Fc (1 μg/ml; BioLegend, catalog no. 410708) at 4°C with gentle shaking in the dark for 1 hour. Cells were washed once and resuspended in ice-cold FACS buffer. Cells were then filtered using a 40-μm cell strainer (VWR) before cell sorting. FACS was performed using a BD FACSAria II cell sorter (BD Biosciences) with a 561-nm laser and a 582/15 band-pass filter. Cells were collected into ice-cold D10 medium [DMEM with glucose (4.5 g/liter), 4 mM l-glutamine, and sodium pyruvate (110 mg/liter; Corning), supplemented with 10% (v/v) FBS (VWR), 1× penicillin-streptomycin (Gibco), and 1× nonessential amino acids (Gibco)] and binned into no (bin 0), low (bin 1), medium (bin 2), and high (bin 3) expression according to PE signal, where each bin contains 25% of the singlet population (fig. S2). A biological replicate of the deep mutational scanning experiment was performed, starting from the transfection step.

### Next-generation sequencing of the NTD mutant library

Sorted cells from each bin were pelleted at 300*g*, 4°C for 15 min and then resuspended in 200 μl of PBS (Corning). Genomic DNA extraction was then performed using the DNA Blood and Tissue Kit (Qiagen, catalog no. 69504) according to the manufacturer’s instructions with a modification: Cells were incubated at 56°C for 30 min instead of 10 min. The NTD mutant library was amplified from the genomic DNA in two nonoverlapping fragments using KOD Hot Start DNA polymerase (MilliporeSigma, catalog no. 710863) per the manufacturer’s instruction with the following two primer sets, respectively (also see table S3): set 1: 5′-CACTCTTTCCCTACACGACGCTCTTCCGATCTCTGCTGCCTCTGGTGTCCAGC-3′ (NTD-DMS-recover-1F) and 5′-GACTGGAGTTCAGACGTGTGCTCTTCCGATCTGTTGGCGCTGCTGTACACCCG-3′ (NTD-DMS-recover-1R); set 2: 5′-CACTCTTTCCCTACACGACGCTCTTCCGATCTAGCTGGATGGAAAGCGAGTTC-3′ (NTD-DMS-recover-2F) and 5′-GACTGGAGTTCAGACGTGTGCTCTTCCGATCTCACGGTGAAGGACTTCAGGGT-3′ (NTD-DMS-recover-2R).

A second round of PCR was carried out to add the adapter sequence and index to the amplicons as described previously (*69*). The final PCR products were submitted for next-generation sequencing using Illumina MiSeq PE300.

### Analysis of next-generation sequencing data

Next-generation sequencing data were obtained in FASTQ format. Forward and reverse reads of each paired-end read were merged by PEAR (*70*). The merged reads were parsed by SeqIO module in BioPython (*71*). Primer sequences were trimmed from the merged reads. Trimmed reads with lengths inconsistent with the expected length were discarded. The trimmed reads were then translated to amino acid sequences, with sequencing error correction performed at the same time as previously described (*40*). Amino acid mutations were called by comparing the translated reads to the WT amino acid sequence. Frequency (*F*) of a mutant *i* at position *s* within bin *n* of replicate *k* was computed for each replicate as follows

$$F_{i,s,n,k}=\frac{{\text{readcount}}_{i,s,n,k}+1}{\sum_{s}\sum_{i}({\text{readcount}}_{i,s,n,k}+1)}$$ (1)

A pseudocount of 1 was added to the read counts of each mutant to avoid division by zero in subsequent steps. We then calculated the total frequency (*F*_total_) of mutant *i* at position *s* as follows

$$F_{\text{total},i,s}=\frac{\sum_{k=1}^{2}\sum_{n=0}^{3}F_{i,s,n,k}}{8}$$ (2)

Mutants with *F*_total_ of equal or greater than 0.0075% were selected for downstream analysis. Subsequently, the weighted average (*W*) of each mutant among 4 bins (bin 0 to bin 3) in each replicate was computed as described previously (*41*)

$$W_{i,s,k}=\frac{F_{i,s,0,k}\;\times \;0.25+F_{i,s,1,k}\;\times \;0.5+F_{i,s,2,k}\;\times \;0.75+F_{i,s,3,k}\;\times \;1}{\sum_{n=0}^{3}F_{i,s,n,k}}$$ (3)

Selected mutants were then categorized based on the mutation types (missense, nonsense, and silent). The mean value of weighted average for nonsense and silent mutations was calculated. Expression score (ES) of a mutant *i* at position *s* of replicate *k* was calculated as described previously (*41*)

$${\text{ES}}_{i,s,k}=\frac{W_{i,s,k}-\bar{W_{\text{nonsense},k}}}{\bar{W_{\text{silent},k}}-\bar{W_{\text{nonsense},k}}}$$ (4)

The final expression score of a mutant *i* at position *s* was calculated by taking the average of the expression scores between replicates. Mutational tolerability of position *s* was then calculated by taking the average of the expression scores of all mutants at that position

$${\text{mutational tolerability}}_{s}=\frac{\Sigma_{i\in s}{\text{ES}}_{i,s}}{\left|\{i\in s\}\right|}$$ (5)

### Structural analysis of deep mutational scanning results

DSSP (*72*, *73*) was used to calculate the solvent exposure surface area (SASA) of each residue in NTD and RBD on the S trimer [Protein Data Bank (PDB) 6ZGE] (*45*). Deep mutational scanning result of RBD was extracted from a previous study (*42*). RSA was computed by dividing the SASA by the theoretical maximum allowed solvent accessibility of the corresponding amino acid (*74*).

Each NTD residue’s distance to RBD/S2 was calculated on the basis of the S trimer structure (PDB 6ZGE) (*45*), with the NTD replaced by the high-resolution crystal structure (PDB 7B62) (*46*). For each NTD residue, the distances to all RBD and S2 residues were measured. The shortest distance was then recorded as the “distance to RBD/S2.” Residue-residue distance was defined as the distance between the centroid coordinates of two residues.

To visualize the mutational tolerability of each NTD residue, the crystal structure of SARS-CoV-2 S protein NTD (PDB 7B62) was used (*46*). The NTD crystal structure was then aligned with the S trimer to generate the figures (PDB 6ZGE) (*45*).

### Evolution analysis of NTD sequences

The sequence conservation analysis of NTD was based on 27 sarbecovirus strains (table S6) (*1*, *75*–*79*). S sequences of these stains were retrieved from GenBank and GISAID (*44*). Their NTD sequences were then identified using tBlastn search using the amino acid sequence of SARS-CoV-2 Hu-1 NTD (gene ID: 43740568) as the query sequence. The BlastXML output of tBlastn was then parsed and used as the input for multiple sequence alignment using MAFFT (*80*, *81*). For each residue position, sequence conservation was defined as the proportion of strains that contains the same amino acid variant as SARS-CoV-2 Hu-1. The information of the 17 SARS-CoV-2 major variants and the observed naturally occurring NTD missense/indel sites were collected from ViralZone (table S7) (*82*).

### Rosetta-based mutagenesis

The structure of the S protein was obtained from PDB (PDB 6ZGE) (*45*). Water molecules and *N*-acetyl-d-glucosamine were removed using PyMOL (Schrödinger). Then, the amino acids were renumbered using pdb-tools (*83*). Fixed backbone point mutagenesis for S50Q and G232E was performed using the “fixbb” application in Rosetta (RosettaCommons). One-hundred poses were generated for each mutagenesis. Using the lowest-scoring structure from fixed backbone mutagenesis as input, a constraint file was obtained using the minimize_with_cst application in Rosetta. Fast relax was then performed via the “relax” application in Rosetta (*47*) with the corresponding constraint file. The lowest-scoring structure out of eight was then used for structural analysis. Code and source files for structural modeling are available in https://github.com/nicwulab/SARS-CoV-2_NTD_DMS/tree/main/rosetta.

### Split mNG2-based cell-cell fusion assay

hACE2 construct was constructed in a previous study (*38*). A split mNG2 reporter system was integrated into the S plasmid (see above) and the hACE2 plasmid (*49*). Specifically, a gene fragment that encoded (from 5′ to 3′) a GCN4 leucine zipper, a GS linker, mNG2_1–10_, and a 2A self-cleaving peptide was inserted into the hACE2 plasmid between the IRES and the hygromycin resistance marker. Similarly, a gene fragment that encodes (from 5′ to 3′) a GCN4 leucine zipper, a GS linker, mNG2_11_, and a 2A self-cleaving peptide was inserted into the S plasmid between the IRES and the puromycin resistance marker. Each plasmid construct was transfected and recombined into HEK293T landing pad cells per step described above.

Once the stable cell lines were created, 5 × 10^5^ landing pad cells expressing hACE2 with mNG2_1–10_ were seeded in six-well plates (Fisher Scientific). The cells were then incubated at 37°C with 5% CO_2_ for 15 min to allow seeding. Subsequently, 5 × 10^5^ landing pad cells expressing S with mNG2_11_ were then added dropwise to the seeded hACE2 cells. Both cells were filtered through 40-μm cell strainer (VWR) before seeding. At 3- and 24-hour postmixing, fusion events in each well were qualitatively assessed with an ECHO Revolve epifluorescence microscope (ECHO) in inverted format. Overlaid images were captured on white light and fluorescein isothiocyanate filter channels using an UPlanFL N 10×/0.30 numerical aperture objective (Olympus) with identical light intensity and exposure settings for all conditions. Cells in each well were then collected using 0.5 mM EDTA, pelleted via centrifugation at 300*g* for 5 min at room temperature, and resuspended in the FACS buffer. LSRII flow cytometry (BD Biosciences) was used to quantify the fusion events of each sample. Negative controls were measured first to set up proper gating strategies (fig. S9). Then, the flow cytometry analysis was performed on 10^5^ live cells for each sample. Data were analyzed using FCS Express 6 software (De Novo Software). The percentage of mNG2-positive population of each sample was used for normalization (table S4).

### Flow cytometry analysis for the protein expression assay and antibody binding assay

Approximately 1 × 10^6^ cells that carried the selected SARS-CoV-2 S NTD mutant were washed with 1× PBS, harvested with warm versene, and pelleted via centrifugation at 300*g* for 5 min at room temperature. The cells were resuspended in the FACS buffer. Subsequently, cells were incubated with 5 μg/ml of the selected antibodies at 4°C with gentle shaking for 1 hour. Cells were then washed once with ice-cold FACS buffer and incubated with PE anti-human IgG Fc (2 μg/ml; BioLegend, catalog no. 410708) at 4°C with gentle shaking in the dark for 1 hour. Cells were washed once, pelleted via centrifugation at 300*g* for 5 min at room temperature, and resuspended in ice-cold FACS buffer. LSRII flow cytometry (BD Biosciences) was used to measure the PE signal of each sample. Negative controls were measured first to set up proper gating strategies (figs. S8 and S10). Then, the flow cytometry analysis was performed on 10^5^ singlets for each sample. Data were analyzed using FCS Express 6 software (De Novo Software).

### Normalization of the expression assay results

The MFI of the entire population was recorded for each sample, followed by the normalization as previously described (*42*). For a given sample *i*, the following equation was used to compute the normalized expression (NE)

$$\text{NE}=\frac{{\text{MFI}}_{i}-{\text{MFI}}_{\text{negative control}}}{{\text{MFI}}_{\text{wildtype}}-{\text{MFI}}_{\text{negative control}}}$$ (6)

Normalizations were performed for each sample within a given biological replicate (table S4).

### Recombinant expression and purification of soluble S protein

SARS-CoV-2 S ectodomain with the PRRA motif in the furin cleavage site deleted and mutations K986P/V987P, which are known to stabilize the prefusion conformation and increase expression (*26*, *48*), was cloned into a phCMV3 vector. The S ectodomain construct contained a trimerization domain and a 6×His-tag at the C terminus. Expi293F cells (Gibco), which were maintained using Expi293 expression medium (Gibco), were used to express soluble S protein. Briefly, 25 μg of the plasmid was transfected into 25 ml of Expi293F cells at 3 × 10^6^ cells ml^−1^ using the ExpiFectamine 293 Transfection Kit (Thermo Fisher Scientific) following the manufacturer’s instructions. Transfected cells were then incubated at 37°C, 8% CO_2_ and shaking at 125 rpm for 6 days. Cell cultures were then harvested and centrifuged at 4000*g* at 4°C for 15 min. The supernatant was clarified using a 0.22-μm polyethersulfone filter (Millipore). S protein in the clarified supernatant was then purified using Nickel Sepharose Excel resin (Cytiva), with 20 mM imidazole in PBS as wash buffer, and 300 mM imidazole in PBS as elution buffer. Three rounds of 2-ml elutions were performed. The eluted protein was then concentrated and analyzed by SDS–polyacrylamide gel electrophoresis reducing gels (Bio-Rad; fig. S6A). Concentrated protein solution was further purified using Superdex 200 XK 16/100 size exclusion column (Cytiva) in 20 mM tris-HCl (pH 8.0) and 150 mM NaCl (fig. S6B). Selected elution fractions were combined and concentrated. Final protein concentration was measured using NanoDrop One (Thermo Fisher Scientific).

### Protein thermostability assay

Five micrograms of purified protein was mixed with 5× SYPRO orange (Thermo Fisher Scientific) in 20 mM tris-HCl (pH 8.0), 150 mM NaCl at a final volume of 25 μl. The sample mixture was then transferred into an optically clear PCR tube (VWR). SYPRO orange fluorescence data in relative fluorescence unit (RFU) was collected from 10° to 95°C using CFX Connect Real-Time PCR Detection System (Bio-Rad). The temperature corresponding to the lowest point of the first derivative, −*d*(RFU)/*dT*, was defined as the melting temperature (*T*_m_). Data were analyzed using OriginPro 2020b (Origin Lab). Raw data are shown in table S5.

## Notes

View/request a protocol for this paper from *Bio-protocol*.
