# Supplementary material for: Probing the biophysical constraints of SARS-CoV-2 spike N-terminal domain using deep mutational scanning
Source: Sci Adv. 2022 Nov 23;8(47):eadd7221. doi: 10.1126/sciadv.add7221 (PMC9683733; doi:10.1126/sciadv.add7221)
Supplement: Supplementary file 1 — Figs. S1 to S10 [file sciadv.add7221_sm.pdf]

Science Advances
AAAS

Supplementary Materials for

Probing the biophysical constraints of SARS-CoV-2 spike N-terminal domain using deep mutational scanning

Wenhao O. Ouyang *et al.*

Corresponding author: Nicholas C. Wu, nicwu@illinois.edu

Sci. Adv. **8**, eadd7221 (2022)
DOI: 10.1126/sciadv.add7221

The PDF file includes:

Figs. S1 to S10
Legends for tables S1 to S7

Other Supplementary Materials for this manuscript includes the following:

Tables S1 to S7

Fig. S1.

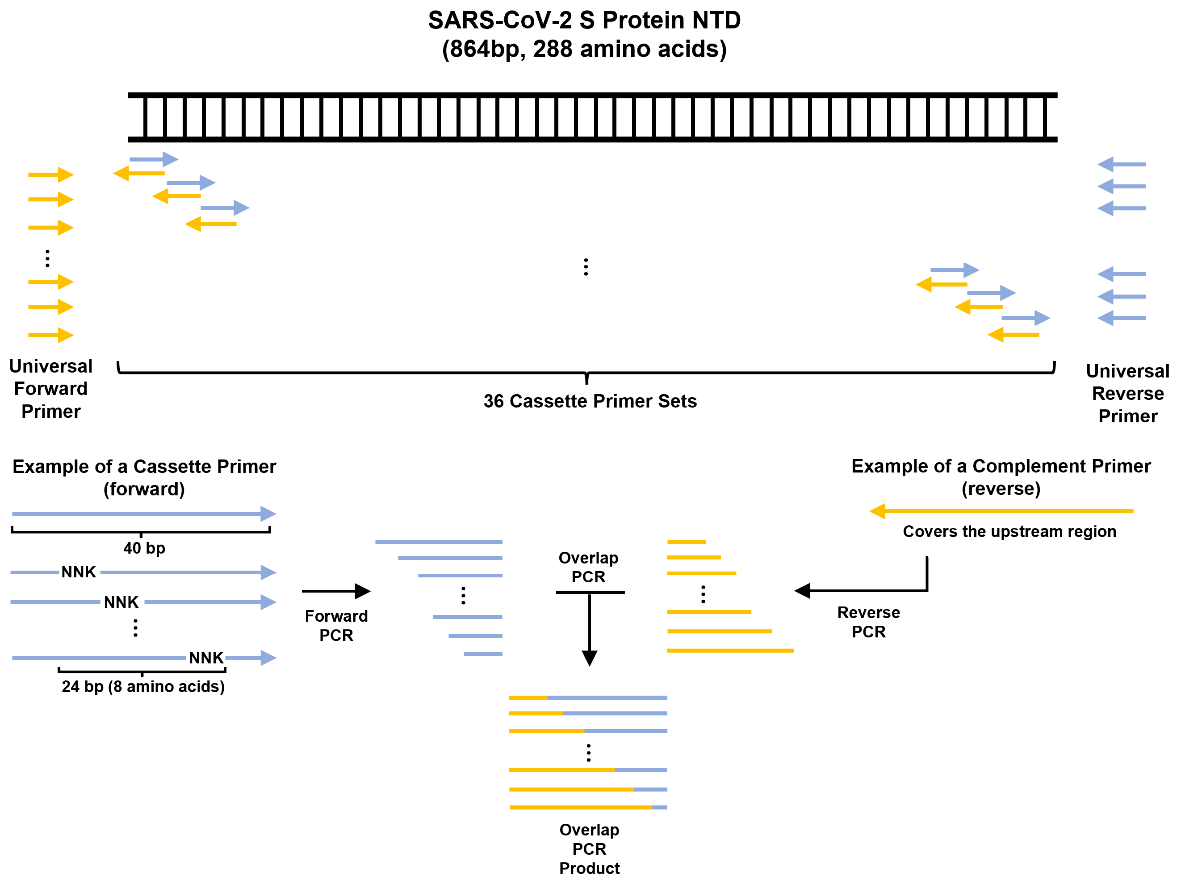

Supplementary Figure 1. Schematics of the cassette-based PCR strategy. Each cassette of primers was used to perform mutagenesis separately. Individual cassette PCRs were pooled together right before restriction enzyme digestion and ligation (see Materials and Methods for more details). This process can prevent potential off-target errors and generation of double mutants.

Fig. S2.

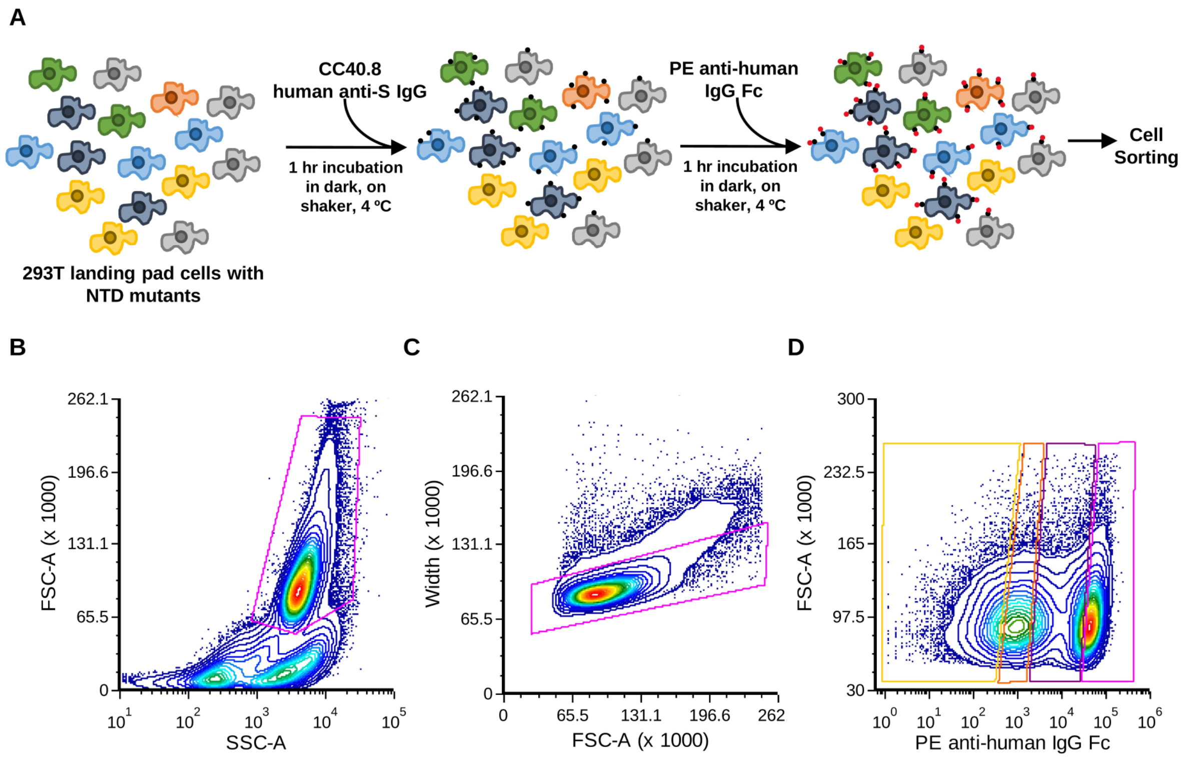

E

Population	Replicate 1 (# of cells)	Replicate 2 (# of cells)
Bin 0 (PE-)	2.303×10^6	2.356×10^6
Bin 1 (PE+)	2.351×10^6	2.324×10^6
Bin 2 (PE++)	2.372×10^6	2.333×10^6
Bin 3 (PE+++)	2.450×10^6	2.328×10^6

Supplementary Figure 2. Overview of the NTD deep mutational scanning workflow. (A) Schematics of NTD deep mutational scanning (see Materials and Methods for details). The black and red dots represent CC40.8 and PE anti-human IgG Fc, respectively. **(B-D)** Gating strategy for FACS is shown. **(B)** Live cells were first gated, then **(C)** the singlets among the live cells were gated, then **(D)** the singlets were sorted into four bins based on the PE signals, each covering 25% of the singlet population. **(E)** Sorting statistics of FACS. Each bin is color coded to correspond each of the four bins shown in **(D)**.

Fig. S3.

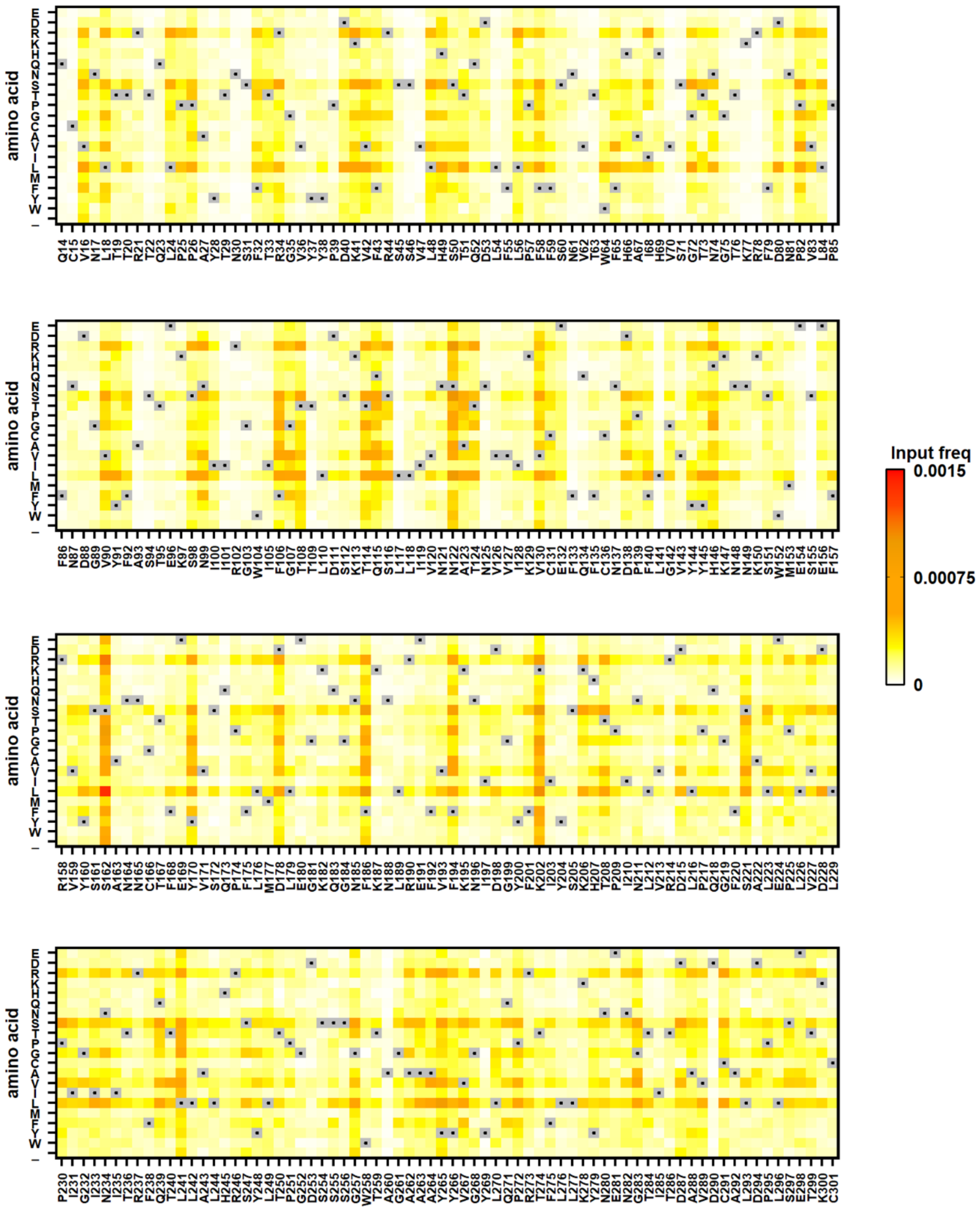

Supplementary Figure 3. Input frequency of each mutant. The input frequencies of individual NTD mutations are shown as a heatmap. X-axis represents the residue position. Y-axis represents different amino acids as well as the stop codon (_). Amino acids corresponding to the WT sequence are indicated by the black dots and shown in grey. It is likely that those NNK codons towards the 3’ end of the cassette primer might lead to poor annealing during the mutagenesis PCR.

Consistently, blocks of low-frequency regions in the heatmap can be observed approximately every eight residues apart, which is concordant with our cassette primer design.

Fig. S4.

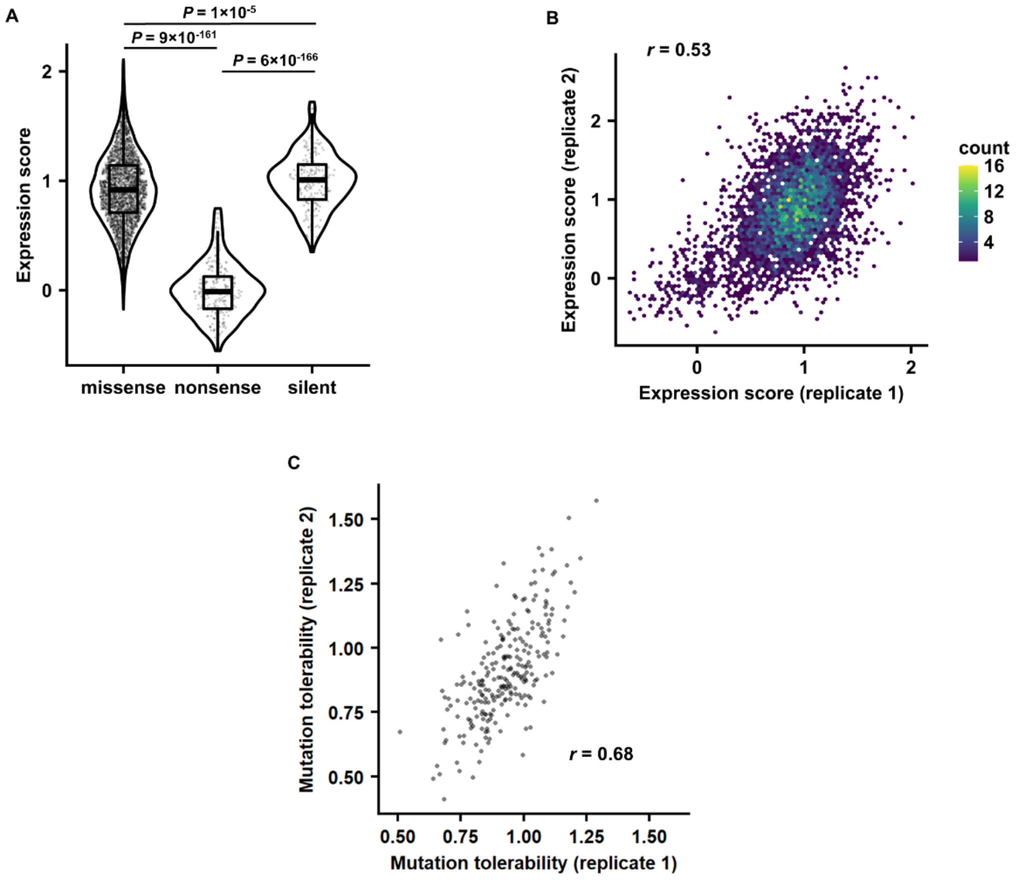

Supplementary Figure 4. Data quality and reproducibility of the NTD deep mutational scanning experiment. (A) The expression score distributions for missense, nonsense, silent mutations are shown as a violin plot. Each datapoint represents one mutation. P-values were computed by two-tailed t-test. **(B)** Correlation of expression scores between two biological replicates is shown as a density scatterplot. **(C)** Correlation of mutational tolerability per site between two biological replicates is shown as a scatterplot. The Pearson correlation coefficient (r) is indicated.

Fig. S5.

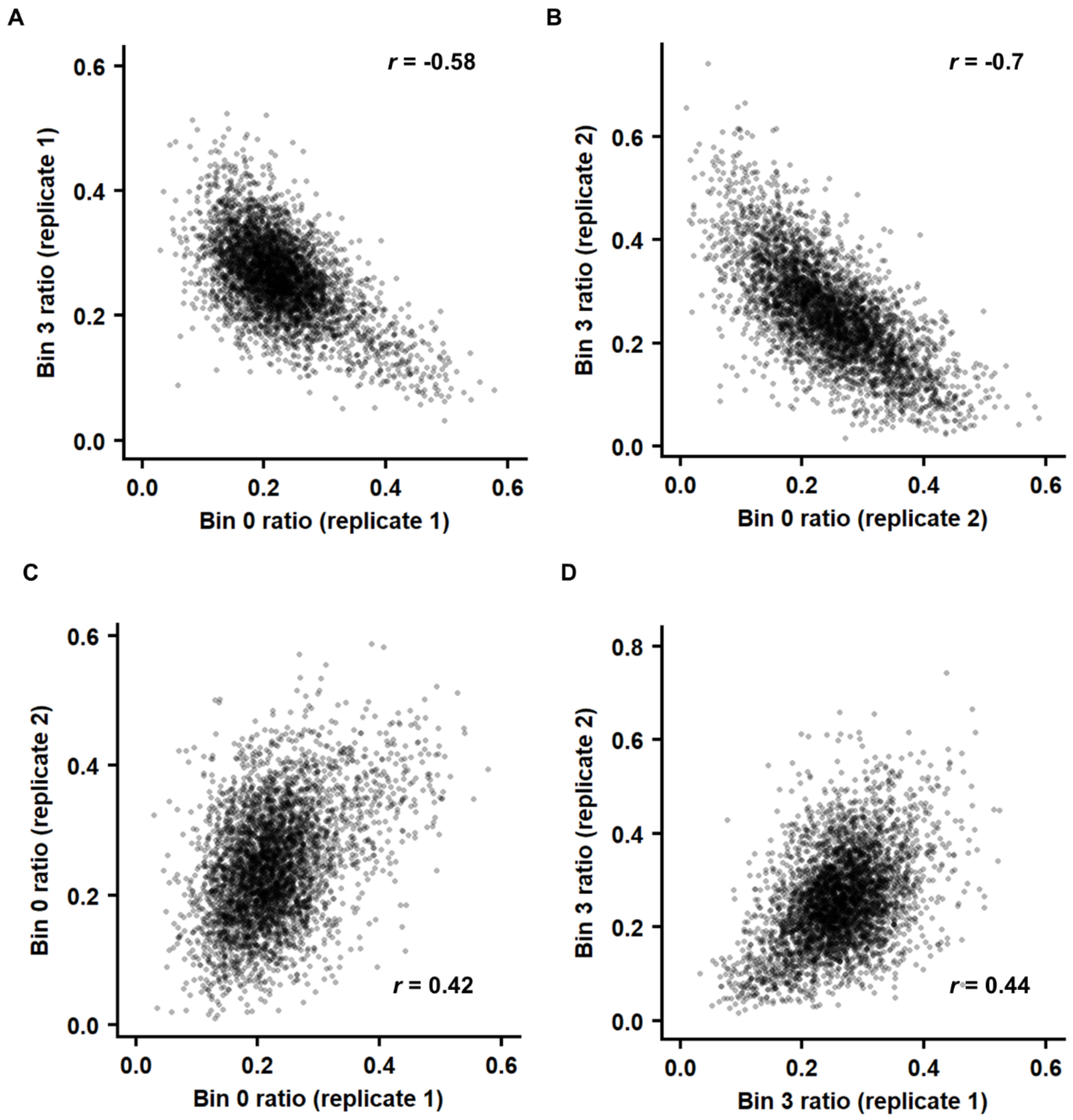

Supplementary Figure 5. Cell viability analysis of the NTD deep mutational scanning experiment. A given mutant is classified as having bimodality if its relative frequency distribution across four bins satisfied the following condition: min(frequency in bin 0, frequency in bin 3) > max(frequency in bin 1, frequency in bin 2). Out of 3999 mutants with nonsense mutations, 349 (8.7%) and 411 (10.3%) exhibited bimodality in replicate 1 and 2, respectively. For each mutant i, we further define its ratio in a given bin n as follow:

$$Ratio_{i,n} = \frac{frequency_{i,n}}{\sum_{bin=0}^{3} frequency_{i,bin}}$$

If cell death was prevalent, the correlation between bin 0 ratio and bin 3 ratio should be minimal. In addition, since cell death should be largely independent from the identity of the S mutant, the correlation between bin 0 from replicate 1 and bin 0 from replicate 2 would also be minimal if cell death was prevalent. However, (A-B) a strong a negative correlation can be seen between bin 0 ratio and bin 3 ratio in both replicates (r = -0.58 and -0.7, respectively), and (C-D) the correlation of bin 0 ratio (r = 0.42) remained similar to the correlation of bin 3 ratio between two replicates (r = 0.44). These observations indicating that cell death did not introduce significant measurement noise in our deep mutational scanning data.

Fig. S6.

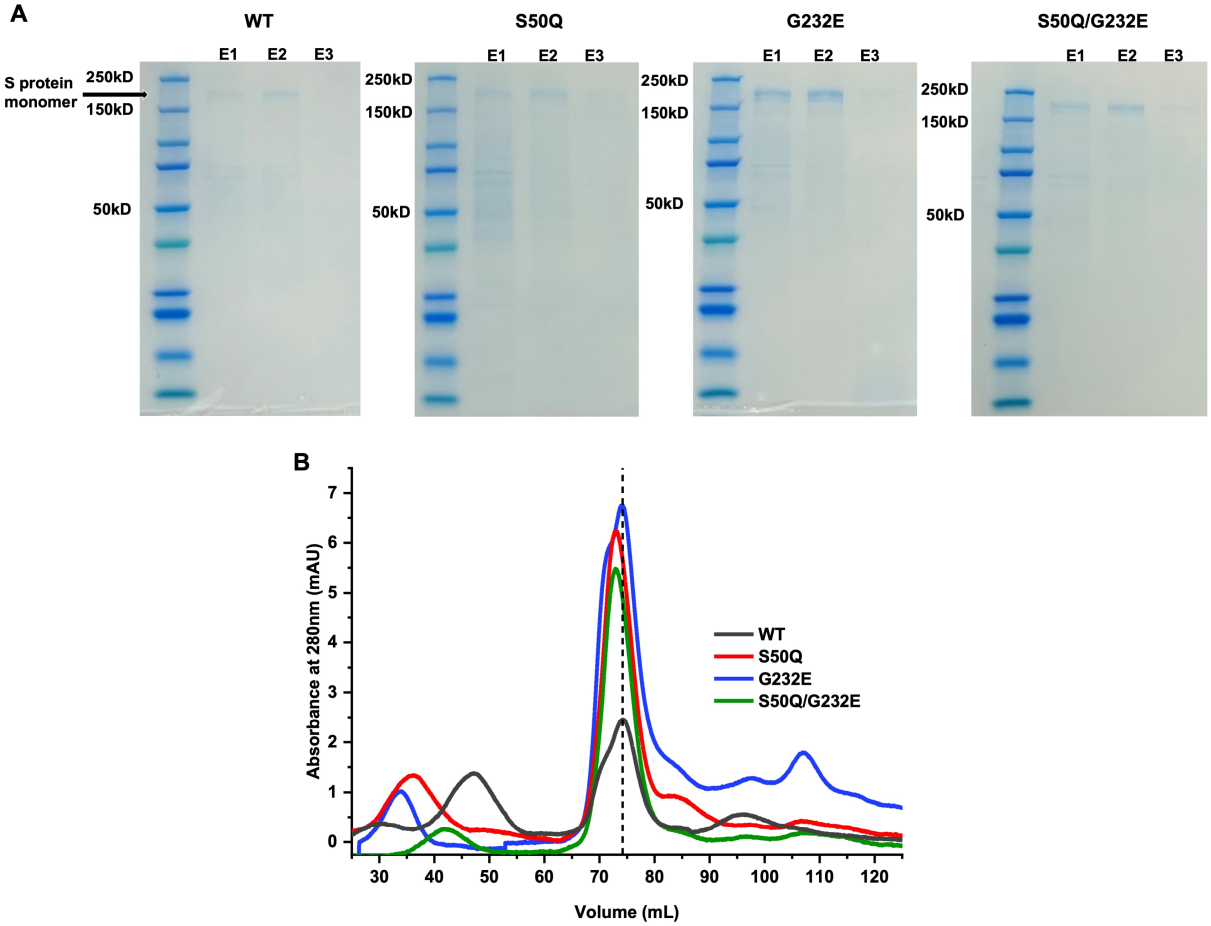

Supplementary Figure 6. Purification and quality control of recombinantly expressed soluble S proteins. (A) The SDS-PAGE gel images of affinity purified WT and mutant soluble S proteins are shown. **(B)** The size exclusion chromatographs of the affinity purified WT and mutant soluble S proteins are compared. The dotted line indicates the retention volume of the WT (74.2 mL).

Fig. S7.

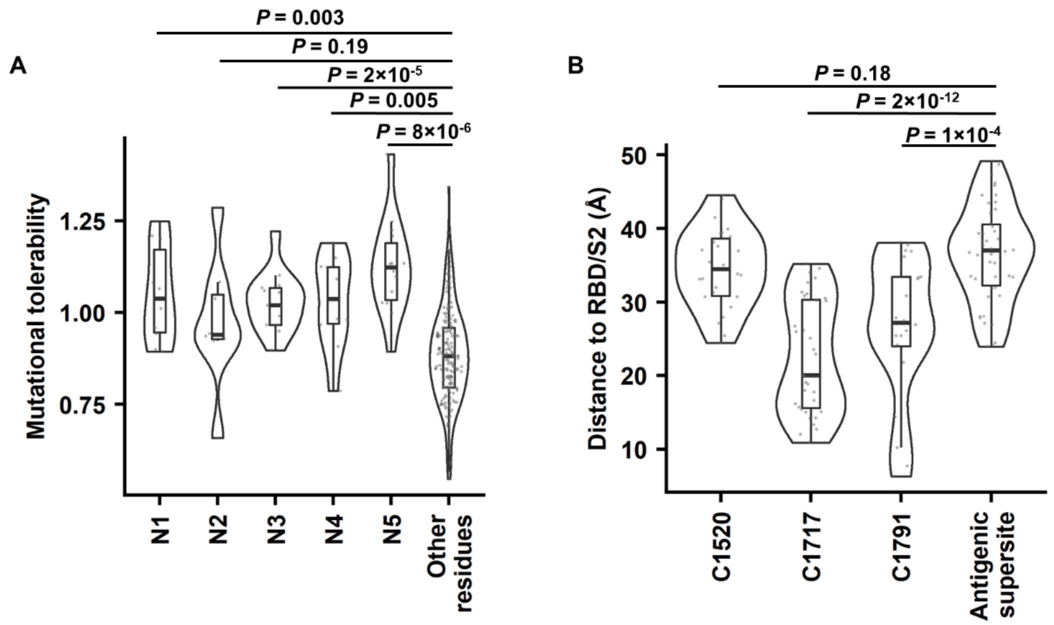

Supplementary Figure 7. Characterization of selected regions on NTD. (A) The difference in mutational tolerability between NTD loop regions (N1-N5) and other NTD residues was illustrated by a violin plot. Each datapoint represents one residue. Regions corresponding to the N1-N5 loops were defined as previously described (*12*). **(B)** The difference in distance to RBD/S2 between epitopes of three cross-neutralizing antibodies (*16*) and the antigenic supersite (*14*) was illustrated by a violin plot. Each datapoint represents one residue. P-values were computed by two-tailed t-test.

Fig. S8.

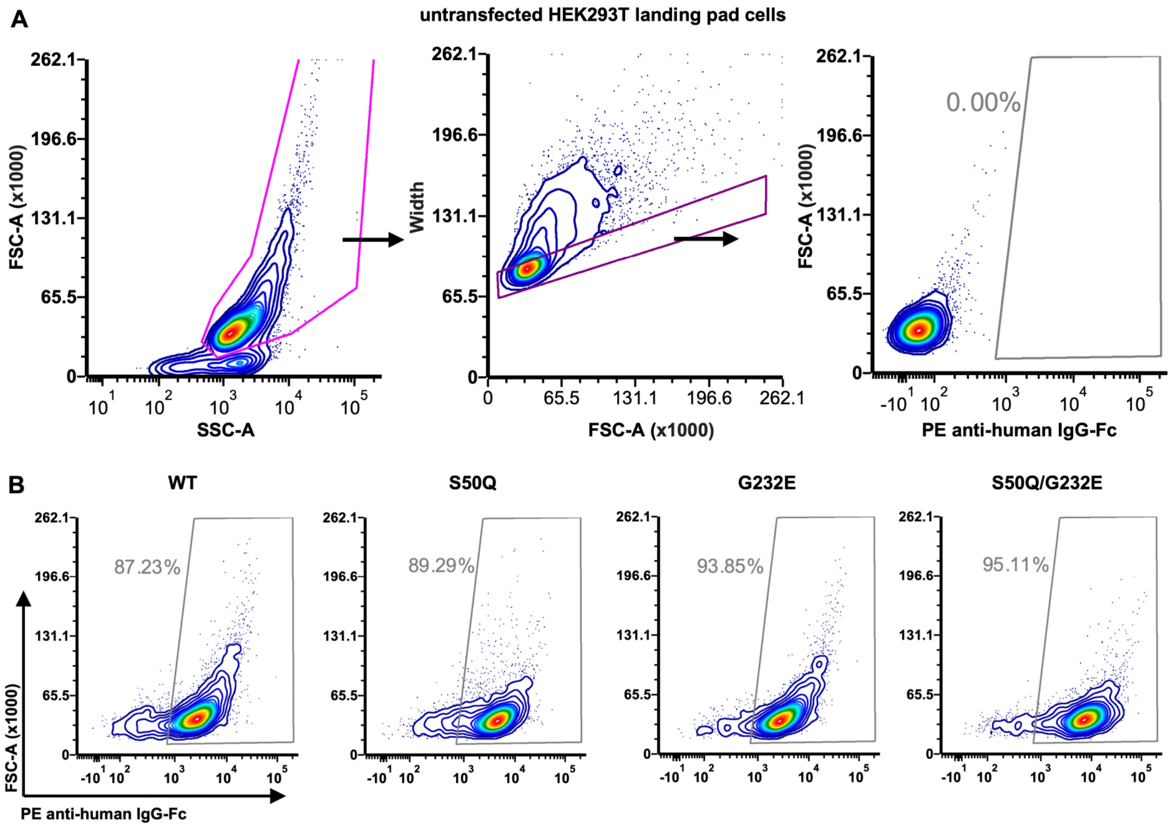

Supplementary Figure 8. Flow cytometry analysis of S protein expression. (A) Gating strategy for measuring the cell surface expression level of WT and mutant S proteins was setup based on untransfected HEK293T landing pad cells, which serve as a negative control. **(B)** Representative flow cytometry results of the WT and mutants.

Fig. S9.

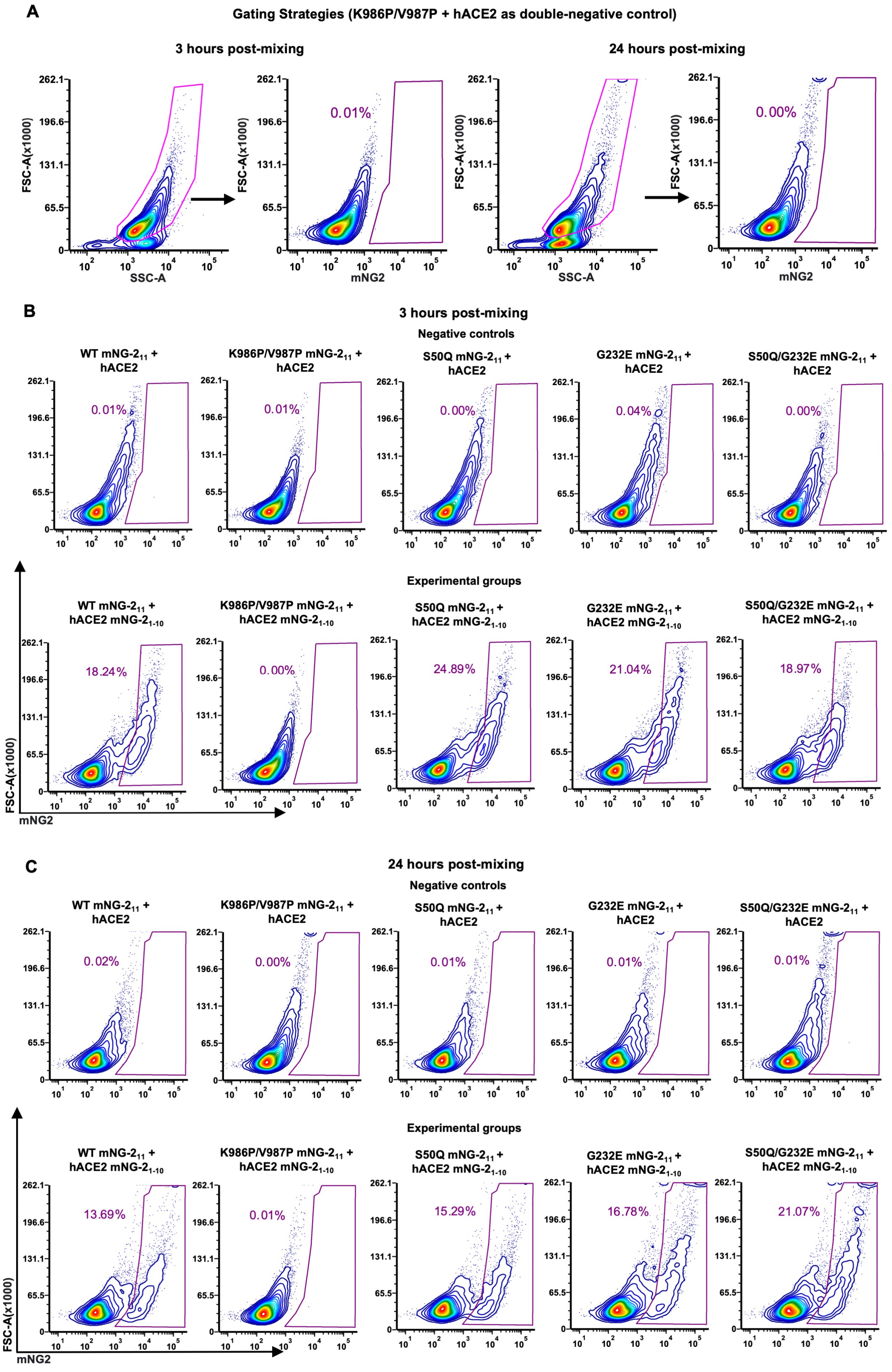
A
Gating Strategies (K986P/V987P + hACE2 as double-negative control)
3 hours post-mixing
24 hours post-mixing
FSC-A(x1000)
SSC-A
mNG2
0.01%
0.00%
B
3 hours post-mixing
Negative controls
WT mNG-2$_{11}$ + hACE2
K986P/V987P mNG-2$_{11}$ + hACE2
S50Q mNG-2$_{11}$ + hACE2
G232E mNG-2$_{11}$ + hACE2
S50Q/G232E mNG-2$_{11}$ + hACE2
0.01%
0.01%
0.00%
0.04%
0.00%
Experimental groups
WT mNG-2$_{11}$ + hACE2 mNG-2$_{1-10}$
K986P/V987P mNG-2$_{11}$ + hACE2 mNG-2$_{1-10}$
S50Q mNG-2$_{11}$ + hACE2 mNG-2$_{1-10}$
G232E mNG-2$_{11}$ + hACE2 mNG-2$_{1-10}$
S50Q/G232E mNG-2$_{11}$ + hACE2 mNG-2$_{1-10}$
18.24%
0.00%
24.89%
21.04%
18.97%
C
24 hours post-mixing
Negative controls
WT mNG-2$_{11}$ + hACE2
K986P/V987P mNG-2$_{11}$ + hACE2
S50Q mNG-2$_{11}$ + hACE2
G232E mNG-2$_{11}$ + hACE2
S50Q/G232E mNG-2$_{11}$ + hACE2
0.02%
0.00%
0.01%
0.01%
0.01%
Experimental groups
WT mNG-2$_{11}$ + hACE2 mNG-2$_{1-10}$
K986P/V987P mNG-2$_{11}$ + hACE2 mNG-2$_{1-10}$
S50Q mNG-2$_{11}$ + hACE2 mNG-2$_{1-10}$
G232E mNG-2$_{11}$ + hACE2 mNG-2$_{1-10}$
S50Q/G232E mNG-2$_{11}$ + hACE2 mNG-2$_{1-10}$
13.69%
0.01%
15.29%
16.78%
21.07%

Supplementary Figure 9. Flow cytometry data evaluating the fusion activity of NTD mutants. **(A)** Gating strategy for the fusion assay was setup based on the mixture of cells that express K986P/V987P S protein (without $mNG2_{11}$) and cells that express hACE2 (without $mNG2_{1-10}$). **(B-C)** Representative flow cytometry results for the fusion assay at **(B)** 3-hour post-mixing, and **(C)** 24-hour post-mixing.

Fig. S10.

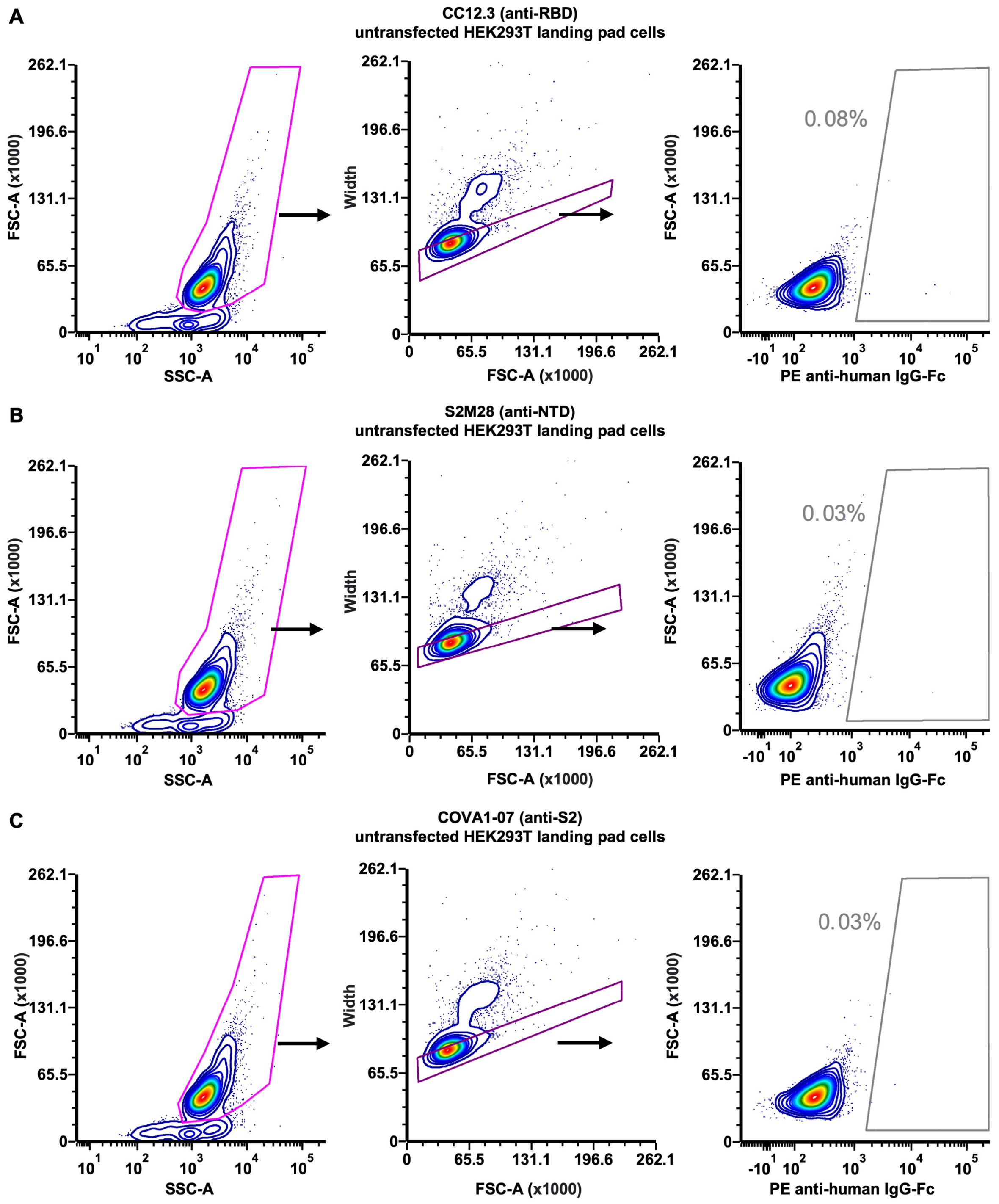

Supplementary Figure 10. Flow cytometry gating strategy for antibody binding experiment. Gating strategy for measuring the binding of S-expressing cells to antibodies **(A)** CC12.3 **(B)** S2M28, and **(C)** COVA1-07 was setup based on untransfected HEK293T landing pad cells, which serve as a negative control.

Table S1. Forward primers for the NTD mutant library construction.

Table S2. Reverse primers for the NTD mutant library construction.

Table S3. Other primers in this study.

Table S4. Experimental data of the expression and fusion assays.

Table S5. Raw data of the thermostability assay.

Table S6. List of the sarbecovirus strains in the sequence conservation analysis.

Table S7. List of the SARS-CoV-2 major variants in naturally circulating mutation analysis
